# Supplementary material for: Toxicity Evaluation of Quantum Dots (ZnS and CdS) Singly and Combined in Zebrafish (Danio rerio)
Source: Int J Environ Res Public Health. 2019 Dec 28;17(1):232. doi: 10.3390/ijerph17010232 (PMC6981874; doi:10.3390/ijerph17010232)
Supplement: Supplementary file 1 [file ijerph-17-00232-s001.pdf]

Table S1. Results from ICP-AES analysis for Zn and Cd in water samples following centrifugation.

Element Analysed (µg/L)		
**Assay**	**Zn**	**Cd**
Control	1.89 ± 0.49	<LOQ
10 µg ZnS/L	4.12 ± 0.89	-
100 µg ZnS/L	70.99 ± 1.85	-
1000 µg ZnS/L	16.34 ± 1.35	-
10 µg CdS/L	-	5.43 ± 0.53
100 µg CdS/L	-	10.60 ± 0.76
1000 µg CdS/L	-	24.51 ± 0.57
10 µg (ZnS + CdS)/L	6.50 ± 0.34	3.44 ± 0.61
100 µg (ZnS + CdS)/L	8.53 ± 1.22	6.44 ± 1.24
1000 µg (ZnS + CdS)/L	24.37 ± 0.70	3.91 ± 0.63

LOD: Cd (0.6 µg/L); Zn (0.3 µg/L). LOQ: Cd (2.0 µg/L); Zn (1.0 µg/L). n = 3
